# Machine learning for hand pose classification from phasic and tonic EMG signals during bimanual activities in virtual reality

**DOI:** 10.3389/fnins.2024.1329411

**Published:** 2024-04-26

**Authors:** Cédric Simar, Martin Colot, Ana-Maria Cebolla, Mathieu Petieau, Guy Cheron, Gianluca Bontempi

**Affiliations:** ^1^Machine Learning Group, Computer Science Department, Université Libre de Bruxelles, Brussels, Belgium; ^2^Laboratory of Neurophysiology and Movement Biomechanics, ULB Neuroscience Institute, Université Libre de Bruxelles, Brussels, Belgium; ^3^Laboratory of Electrophysiology, Université de Mons-Hainaut, Mons, Belgium

**Keywords:** EMG, classification, hand gestures, machine learning, xDAWN filtering, neural integrator, data acquisition

## Abstract

Myoelectric prostheses have recently shown significant promise for restoring hand function in individuals with upper limb loss or deficiencies, driven by advances in machine learning and increasingly accessible bioelectrical signal acquisition devices. Here, we first introduce and validate a novel experimental paradigm using a virtual reality headset equipped with hand-tracking capabilities to facilitate the recordings of synchronized EMG signals and hand pose estimation. Using both the phasic and tonic EMG components of data acquired through the proposed paradigm, we compare hand gesture classification pipelines based on standard signal processing features, convolutional neural networks, and covariance matrices with Riemannian geometry computed from raw or xDAWN-filtered EMG signals. We demonstrate the performance of the latter for gesture classification using EMG signals. We further hypothesize that introducing physiological knowledge in machine learning models will enhance their performances, leading to better myoelectric prosthesis control. We demonstrate the potential of this approach by using the neurophysiological integration of the “move command" to better separate the phasic and tonic components of the EMG signals, significantly improving the performance of sustained posture recognition. These results pave the way for the development of new cutting-edge machine learning techniques, likely refined by neurophysiology, that will further improve the decoding of real-time natural gestures and, ultimately, the control of myoelectric prostheses.

## 1 Introduction

Myoelectric prostheses have emerged in recent years (Chen et al., 2023) as an increasingly promising tool for enhancing natural hand function restoration in individuals with upper limb loss or deficiencies. This has been made possible by significant advances in machine learning and increased accessibility of bioelectrical signal acquisition devices and miniaturized computing units. Myoelectric prostheses typically use surface electromyography (sEMG), a non-invasive physiological measurement technique that records the electrical activity produced by the activation of motor units within muscle tissues during voluntary muscle contractions. When a muscle is voluntarily contracted, motor neurons in the spinal cord are progressively recruited, leading to the activation of motor units and their innervated muscle fibers within the target muscle (Godaux and Chéron, 1989). This electrical activity is captured by specialized electrodes placed on the surface of the skin and transmitted to the myoelectric prosthesis which decodes and replicates the user's intended movements (Nguyen et al., 2021).

The design, control, and functional capabilities of myoelectric prostheses for upper limb amputees are influenced by the level of amputation and the availability of residual muscles for recording specific myographic activity. Transhumeral amputation, occurring above the elbow and involving the loss of both the forearm and hand, requires a prosthetic arm to enable elbow movement and provide basic hand functions. In this situation, a myoelectric prosthesis typically has fewer degrees of freedom (DOFs), focusing on forearm rotation (pronation/supination) and hand grasping and releasing. For transradial amputation, occurring below the elbow joint, more residual muscles are typically available, allowing myoelectric prostheses to additionally restore finer hand functions such as pinching or single-finger control.

From the 1960th, neuroscience has been benefiting from engineering science, especially in the field of motor control (Stark, 1968). For instance, the incorporation of mathematical integrators into the study of oculomotricity led to the recognition of neural integrators within the brain (Robinson, 1968; Chéron et al., 1986; Cannon and Robinson, 1987). This concept has been widely accepted and is also considered valid in the broader context of overall motor control. However, we propose that the development of human assist devices today should be drawing from the foundations of neuroscience in motor control. Specifically, machine learning models should leverage both theoretical and practical neurophysiological advancements to further optimize the extraction of discriminative characteristics from the biological signals at the foundation of human movement (Thomas et al., 2023).

When producing a movement and holding the resulting posture, the EMG signal typically comprises a phasic and a tonic component. The phasic component represents the dynamic bursts of electrical activity produced by the rapid firing of motor units within the muscle during contractions and movement execution (Winges et al., 2013). In contrast, the tonic component represents the relatively constant and lower-intensity electrical activity in the muscle when sustaining the resulting posture. While some authors proposed that EMG patterns offer an accurate reflection of the motor program used by the central nervous system (CNS) for movement control (Gottlieb, 1993), others argue that both EMG and kinematic patterns emerge as non-programmable properties of the system. According to this second perspective, control signals inherently possess positional information of the limb rather than muscle activation necessary to reach the target position (McIntyre and Bizzi, 1993; Feldman et al., 1998; Gribble et al., 1998). Although phasic and tonic components both represent observable peripheral outcomes of CNS control signals, they exhibit distinct physiological characteristics (Flanders and Soechting, 1990; Buneo et al., 1997). Therefore, differentiating between inertial and postural activities can enhance the ability of classification or regression models to identify mapping relationships between EMG signals and limb trajectories during complex movements (Draye et al., 2002; Phataraphruk et al., 2022).

Recent literature extensively covers the use of EMG classification pipelines to discriminate predefined discrete or simultaneous movements (Young et al., 2014) for sign language recognition (Savur and Sahin, 2015; Ben Haj Amor et al., 2023) or hand gesture recognition for prosthesis control (Jaramillo-Yánez et al., 2020). In these scenarios, the recorded EMG activity is processed by a classification pipeline, which identifies the corresponding predefined gesture and subsequently actuates the prosthesis to replicate it. In the present study, we introduce a novel experimental paradigm leveraging a consumer-grade virtual reality (VR) headset equipped with hand-tracking capabilities. This technology enables us to precisely impose visually-controlled and reproducible hand positions and allows us to track and monitor the representation of VR movements. We recorded synchronized EMG signals and full hand pose estimations from 14 healthy participants using two distinct acquisition protocols in virtual reality. The first protocol focused on the execution of predefined bimanual gestures, while the second protocol recorded unconstrained bimanual movements across various virtual scenarios. We expect the recording of both EMG signals coupled with articular kinematics of the hand in both protocols to enable the classification of specific predefined gestures, as commonly explored in existing literature. But more importantly, we aim at approaching more effective real-time regression through the use of 51 degrees of freedom corresponding to the articulation angles of the human hand. This could lead to achieving significantly finer, more reactive, and more natural control of future hand prostheses.

Here, we first validate this novel experimental paradigm through a comprehensive analysis of the resulting EMG signals. Next, we compare multiple classification pipelines based on standard signal processing features, convolutional neural networks, and covariance matrices with Riemannian geometry and for intra-subjects, inter-sessions, and inter-subjects gesture classification. We demonstrate the performance of the latter for gesture classification, achieving 96.9%, 86.8%, and 73.9% accuracy using the phasic component, and 98.3%, 82.3%, and 68.9% accuracy using the tonic component in intra-subjects, inter-sessions, and inter-subjects configurations, respectively. This study comprehensively assesses the discriminative power of the phasic and tonic components of movement in the context of classifying predefined gestures. Interestingly, we show that the long-lasting tonic component of an EMG signal during a sustained gesture contains at least as much discriminative power as the dynamic bursts of the phasic component during the execution of the movement. This result demonstrates that robust continuous recognition of a desired posture can rely on the long-lasting tonic component, without any loss of discriminative power when compared to the short-lasting phasic component. These results could prove useful for future studies on prosthesis control. Finally, we show that integrating physiological knowledge into signal-processing techniques improved classification performance in specific scenarios. This paves the way for broader applications of fundamental neurophysiological methods to further enhance state-of-the-art discrimination of EMG signals and human-machine interfaces.

## 2 Materials and methods

### 2.1 Participants

Data were collected from 14 healthy volunteers (31.7 ± 12.9 years old). 13 volunteers were right-handed and one volunteer was left-handed [determined by the Handedness inventory (Oldfield, 1971)]. All participants presented no neurological condition, and normal vision, including 3D vision. More information about the participants is given in Table 1. All experimental protocols were approved by the Ethics Committee of Université Libre de Bruxelles, CHU Brugmann under the reference CE 2023/37 and conducted in conformity with the European Union directive 2001/20/EC of the European Parliament.

**Table 1:** Information on the participants.

**Participant**	**Age**	**Leading hand**	**Gender**
1	55	Right	M
2	34	Right	M
3	29	Right	F
4	22	Right	M
5	31	Right	M
6	26	Right	M
7	22	Right	M
8	34	Left	M
9	29	Right	M
10	22	Right	M
11	34	Right	M
12	20	Right	F
13	20	Right	F
14	66	Right	F

### 2.2 Experimental design

#### 2.2.1 EMG data collection

**Figure 1:**
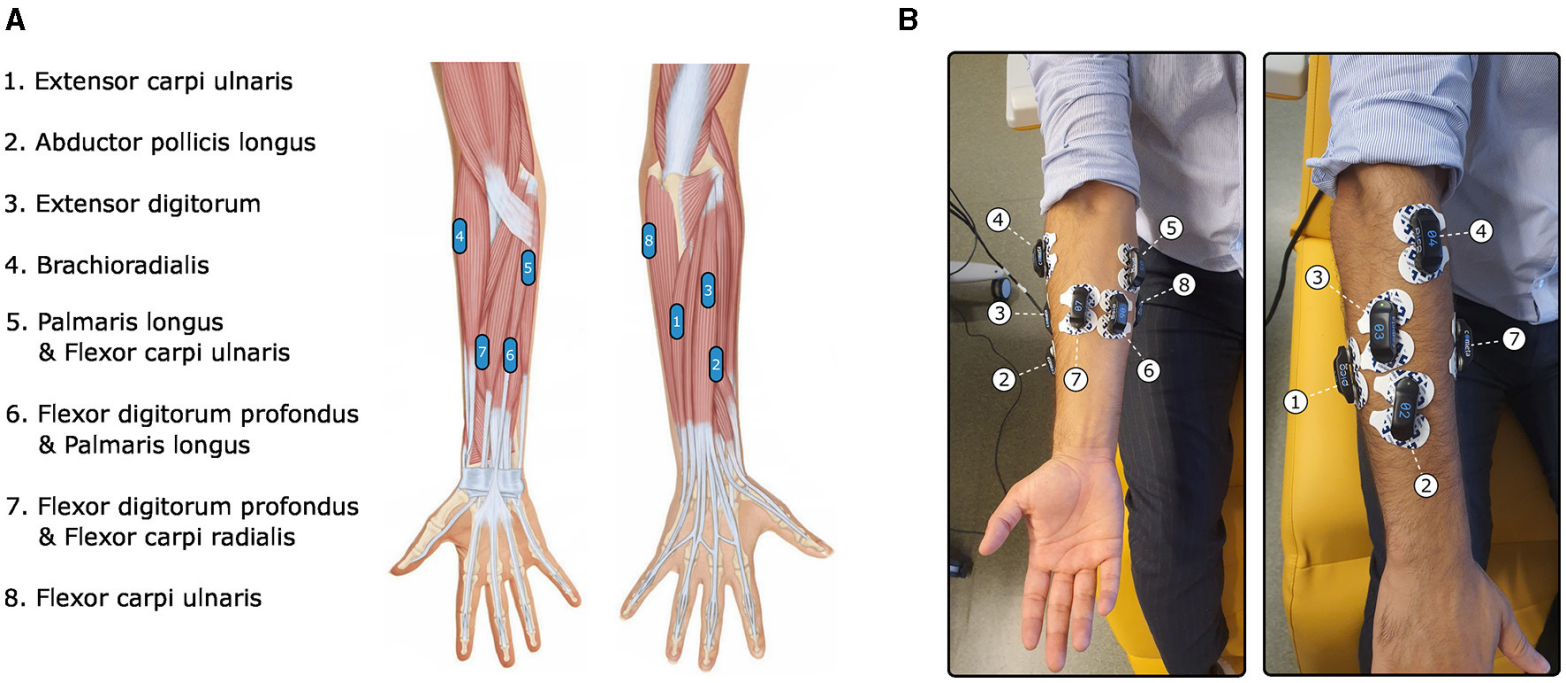
Illustrations of the EMG electrodes placement on a right arm. **(A)** Anatomical illustration of the electrodes locations. **(B)** Electrodes placement on a participant's arm.

EMG signals from the left and right forearms were collected using 16 wireless picoEMG sensors manufactured by Cometa, with a sampling frequency of 2,000 Hz. These sensors are wireless and lightweight, can be worn comfortably and only require the adhesive attachment of two pre-gelled electrodes before use. Synchronized EMG data were recorded through a dedicated computer linked to the 16 sensors via the Cometa wifi router. The placement of the 16 EMG sensors followed a symmetrical pattern on both forearms, as illustrated in Figure 1. Sensor locations were determined by trained neurophysiologists using palpation (Hioki and Kawasaki, 2009) and electrical stimulation capable of eliciting flexion or extension of the fingers.

#### 2.2.2 Motion capture data collection

Traditional motion capture techniques are often costly and complex. The widely used 3D motion camera tracking systems with markers require a high level of expertise to be set up for precise tracking of hand movements. To collect kinematic data from both hands, we used an Oculus Quest virtual reality headset^1^ as a cost-effective alternative with reliable hand-tracking capabilities. This device is equipped with four infrared cameras and uses computer vision to estimate hand and finger movements in real-time. We developed a dedicated software using the Unity framework^2^ and the *OVR* library^3^ to record hand gestures at a sampling frequency of 50 Hz. We synchronized the Oculus Quest hand motion capture alongside physiological data. The application of an Oculus Quest for hand motion capture alongside physiological data is a novel approach which will be validated in this study. This approach enables the complete immersion of the participant within a fully monitored and controllable virtual reality environment. In this setting, participants were instructed to either execute predefined hand gestures or to engage in natural interactions with objects in the virtual surroundings.

Figure 4B illustrates the virtual hand mechanical model. For each joint, the *OVR* library computes a 3D articular rotation, relative to the parent joint. The rotation values of the 17 joints are used to automatically recognize several predefined postures. We use the recognized posture as a label when creating the samples for our classification pipelines. During guided gesture exercises, we also labeled the samples with the posture that the subject was asked to perform.

For each session of each participant, we observed that the Oculus Quest consistently recognized all the predefined gestures introduced in Section 2.2.4.1. The alignment of the kinematics with the prime mover EMG spike was also verified independently. Additionally, since the Oculus Quest captures the hand gestures at a frequency of 50 Hz, we can theoretically expect a maximum variability of 20 ms.

#### 2.2.3 Experimental setup

**Figure 2:**
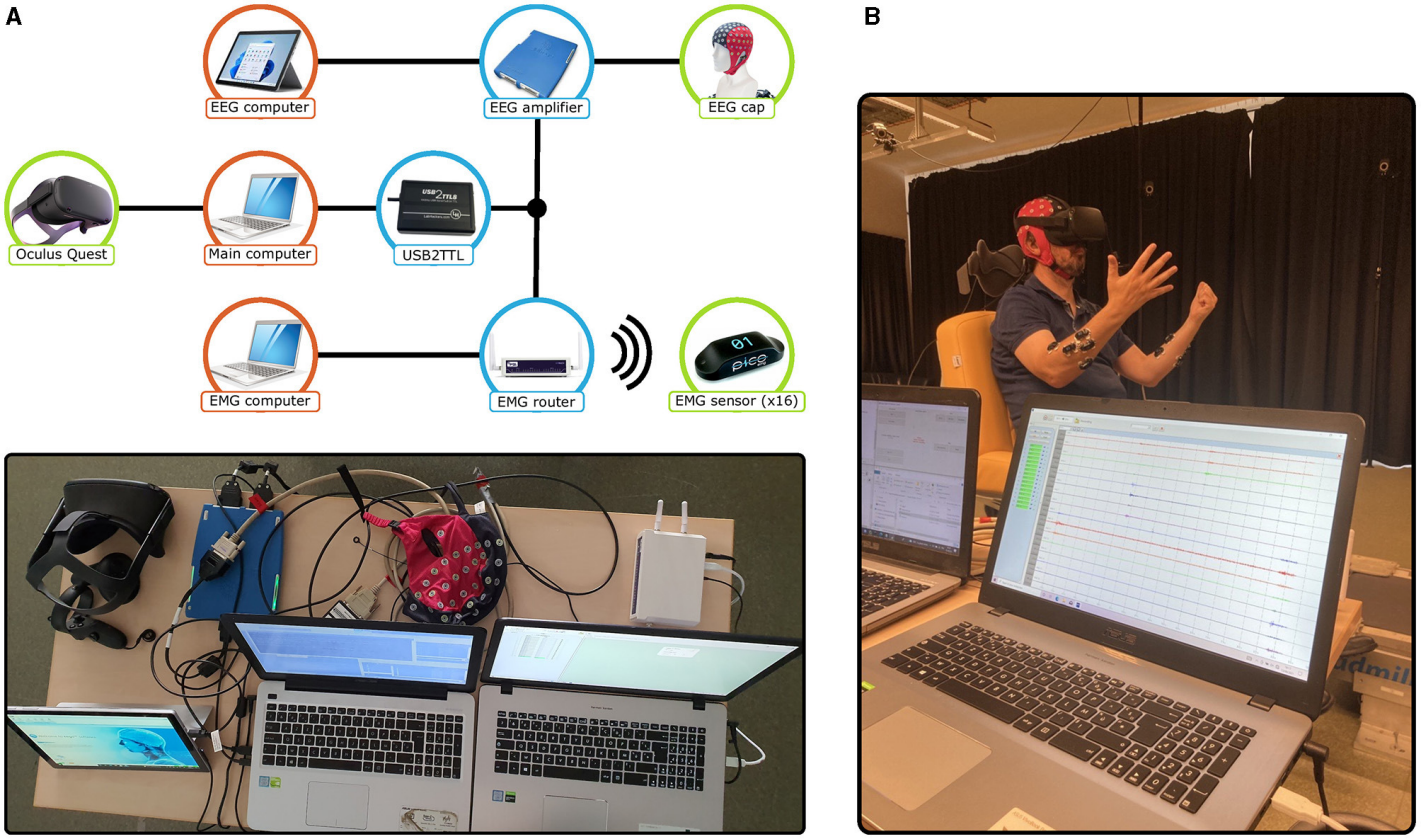
**(A)** Diagram and picture of the experimental setup. The diagram features the monitoring computers in red, the acquisition devices in green, and the connection components in blue. **(B)** Picture of a participant during a recording session of guided gestures.

The experimental setup is composed of the Oculus Quest, for motion capture, the EMG sensors, and a 64-channel ANTneuro EEGO sport EEG cap (not used in the present analysis). The acquisition setup includes three specialized computers that monitor the entire process. Figure 2 illustrates how these components are connected. With the main computer as the central hub, it is possible to send precise trigger signals for initiating the EMG recording and adding annotations to the EEG recording. A wire connection between the main computer and the Oculus Quest enables the display of the different exercises and the start of the motion capture recordings. By initially aligning the UTC timestamps from the main computer and the Oculus Quest, data synchronization was established. Verification of every recording was subsequently thoroughly performed through manual visual inspection.

#### 2.2.4 Data acquisition protocol

Participants were asked to come to the laboratory for four consecutive hours. After being sat comfortably in a chair, the purpose of the experiment was explained and the participant was equipped. In order to facilitate the comparison between different muscle activity across different participants, a normalization was realized by taking into account the absolute value of the EMG amplitude recorded during Maximum Voluntary Contractions (MVC) (Lehman and McGill, 1999). MVC was performed using three three-second isometric exercises using both hands simultaneously: finger flexion, finger extension, and clenching of the fists against a resisting force (Dahlqvist et al., 2018). Afterward, the participants were asked to execute two types of exercises: guided gestures and free gestures. Each participant performs 11 sessions, each session taking about 5 min. The recording starts with a free gesture session, and alternates between guided gestures and free gestures. At the end, a total of five guided exercises and six free exercise sessions are completed by the participant. Then, the equipment was removed and the participant was debriefed. In the present study, we analyzed only the guided gestures.

##### 2.2.4.1 Guided exercises

**Figure 3:**
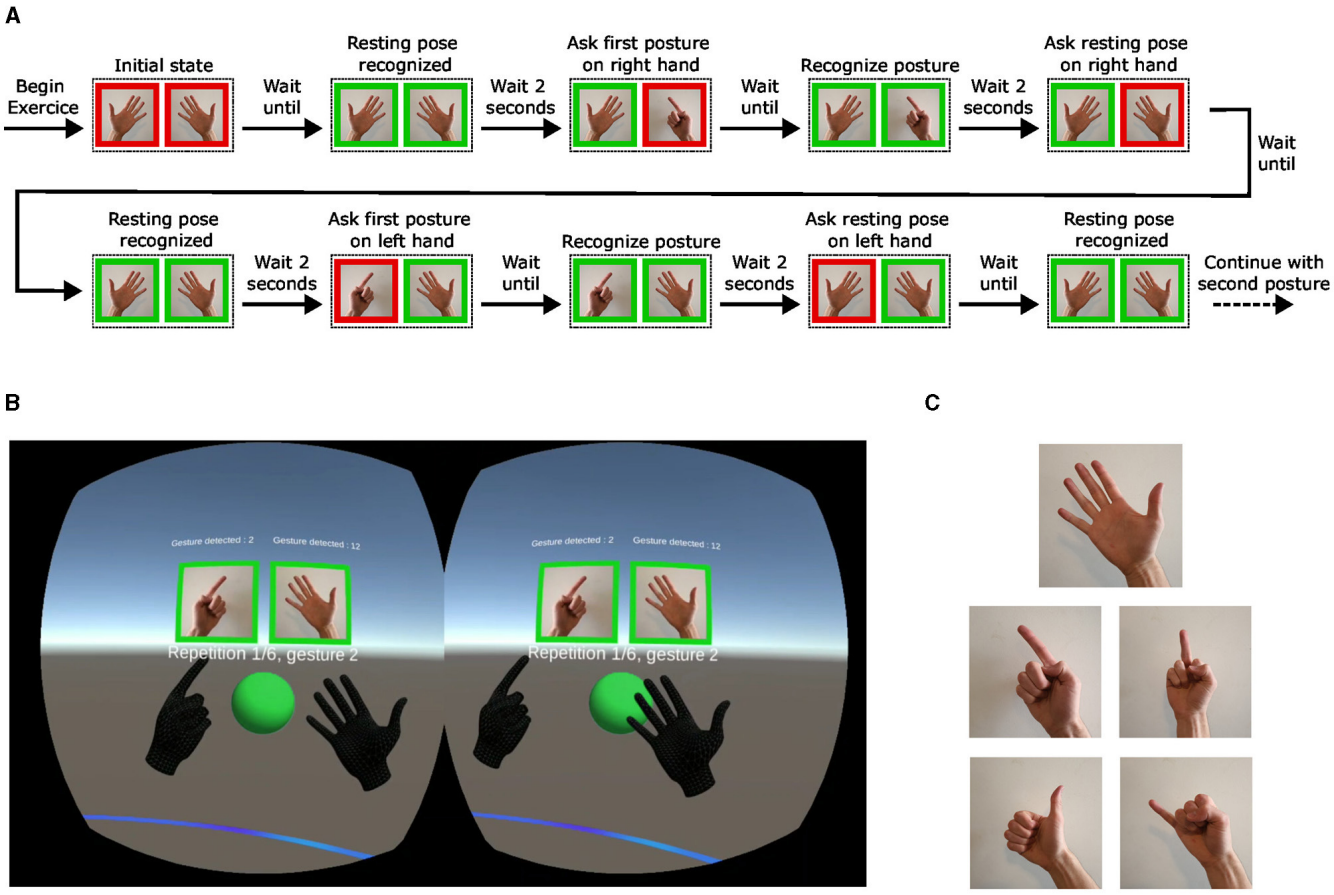
**(A)** Experimental protocol for predefined gestures. **(B)** View of the VR hands and user interface from inside the Oculus Quest. **(C)** Pictures of the five guided gestures recorded during guided exercises, including the open hand as the resting pose.

**Figure 4:**
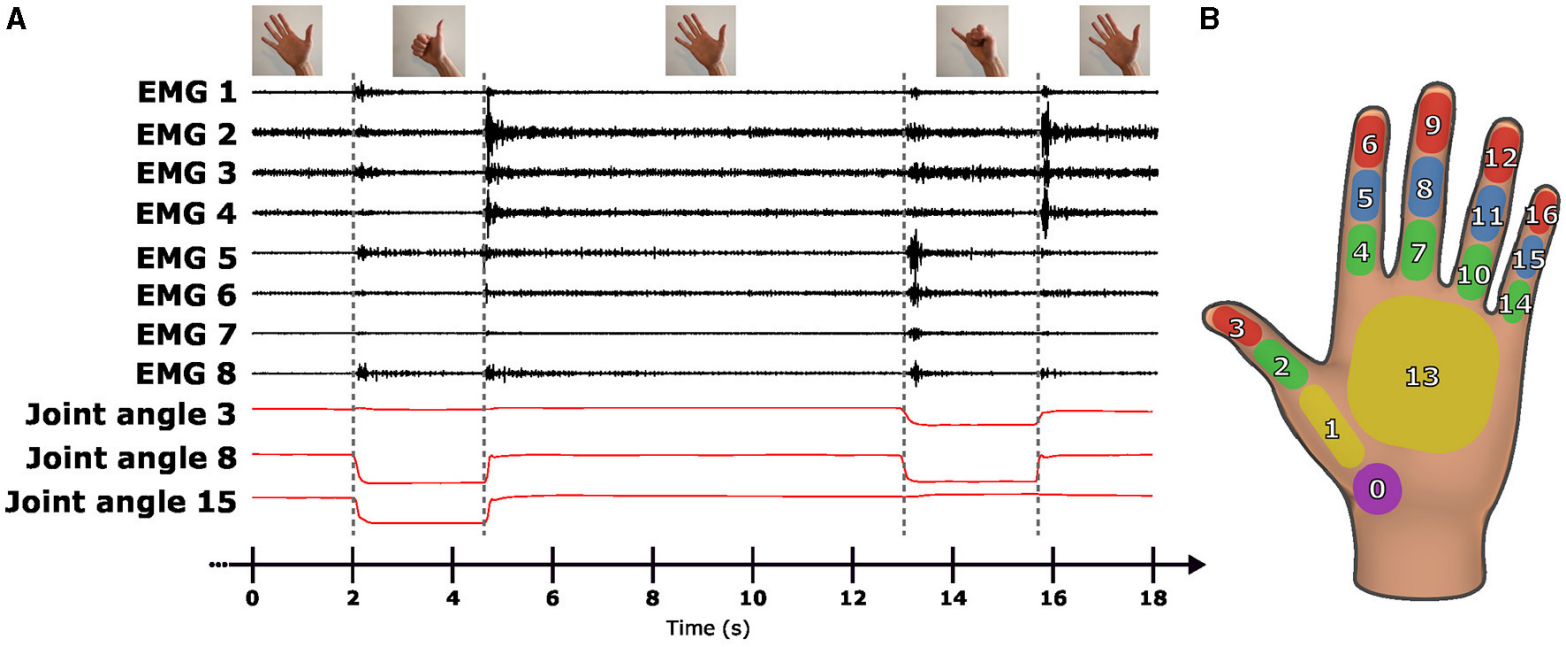
**(A)** A section of the raw EMG signal (in black) and the evolution of three joint angles (in red) from one participant during a guided exercise. **(B)** The mechanical model of the hand displayed in the Oculus Quest.

The recording of guided gestures involves participants alternating between a resting pose (an open hand) and four different finger postures, all while keeping both hands in front of them. Each of those postures (shown in Figure 3C) consisted in the extension of the thumb, index, middle, and pinky fingers. The user interface of the Oculus Quest is used to display the target posture to the participant, as shown in Figure 3B. When the picture of the target posture changes, a red frame is displayed, indicating the update of the target posture. When the Oculus Quest recognizes that the target posture has been successfully performed, this frame becomes green and the participant is asked to hold the pose for two seconds. This real-time recognition is automatically performed by the Oculus Quest, which continuously maps hand motion capture to predefined patterns integrated into the Oculus Quest. The diagram in Figure 3A shows the interface evolution during the execution of one guided gesture with the two hands. Each posture is performed six times during the exercise in a random order established at the beginning of every session. At the end of the recordings, a participant performed 30 times each gesture with both hands. An illustration of the EMG signals recorded on the right forearm is shown in Figure 4A, aligned with three representative signals of articular angles during this exercise. The recording protocol for the free gestures sessions is not described here as the data acquired during this exercise is not analyzed in the present paper.

### 2.3 EMG and hand motion capture data treatment

After removing data segments recorded before the first rectangular pulse of the synchronization signal, the raw EMG and hand motion capture data recordings were resampled at 2,048 Hz and encapsulated into a common data structure using the MNE-Python library (Gramfort et al., 2013; Larson et al., 2023). Subsequently, EMG signals were processed by applying a notch filter with a central frequency of 50Hz and a bandwidth of 5Hz to eliminate power line interference and using a zero-phase IIR bandpass filter with cutoff frequencies set at 30 and 500 Hz to mitigate lower-frequency baseline drift and higher-frequency noise. Finally, for each participant, the quality of EMG signals and their synchronization with hand motion capture data were validated through careful visual inspection.

Subsequently, the EMG signals from each subject were normalized by dividing each electrode's EMG signal by the maximum absolute value recorded for that electrode during MVC recordings. This method has been demonstrated to be effective for achieving robust inter-subject normalization (Burden and Bartlett, 1999). Normalizing the EMG from MVC is expected to provide better results than setting the maximum EMG amplitude to one because MVC records are designed to contain the real maximum amplitude of the signal. It also enables the normalization of the samples in real time without having to wait for all the samples to be collected.

For real-world applications, it is necessary to recognize the hand gestures in real-time (i.e. making multiple posture estimation per second). We make a first step in this direction by analyzing small windows of EMG signal separately. Consequently, for a single repetition of a gesture, we obtain several windows containing different parts of the signal. During the motion of the limb, the window contains phasic components, and during the hold of the limb at the target posture, the window contains tonic components. In this work, we separate the two kinds of epochs to assess their respective predictive power.

We separate the phasic and tonic samples by building two datasets from each participant's guided session one using the phasic component and another using the tonic component. For the dataset using the phasic component of the EMG signals, we extracted epochs of 500 ms before the signs were recognized by the VR headset. As illustrated in Figures 4, 7, this timeframe effectively encompasses the whole phasic component of the physiological movement. For the dataset using the tonic component of the EMG signals, we extracted overlapping epochs of 500 ms with a 256 ms stride, starting at 500 ms and extending to 2,000 ms after the sign was recognized by the VR headset.

Also, despite our best efforts, a visual inspection of the EMG signal by experts showed that a limited number of the recorded sessions had EMG signals contaminated by artifacts caused by low electrode battery levels or poor skin contact with the participant. Those artifacts are characterized by repeated sharp changes in the amplitude of the EMG. In the dataset, we solely included sessions with the best signal's quality to compute pipelines performances. We thus discarded eight sessions on 70 (11.4%) from the original recordings, marked as containing low-quality signals from at least one electrode. The phasic and tonic datasets contain 1,472 and 7,362 data points, respectively, each with a shape of 8 × 512.

### 2.4 Classification pipelines

**Figure 5:**
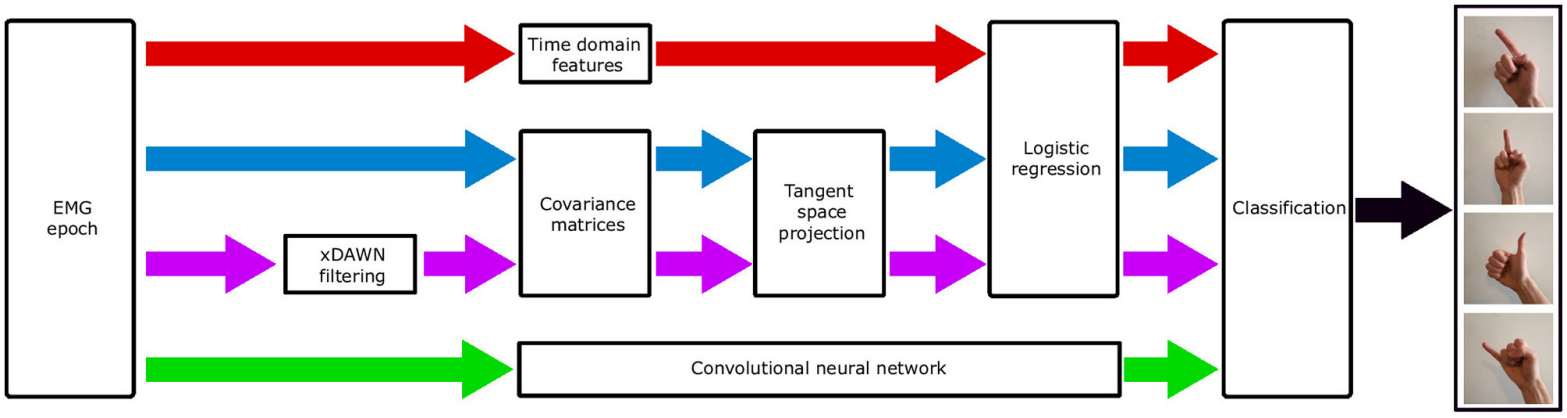
Illustrative summary of the four classification pipelines. The first pipeline, shown in red, uses standard time domain features and a logistic regression. The second pipeline, shown in blue, uses covariance matrices, projection in the tangent space, and a logistic regression. The third pipeline, shown in purple, extends the second by introducing xDAWN spatial filtering. The fourth pipeline uses a convolutional neural network, acting as both a feature extractor and classifier.

We here formally introduce four classifications pipelines whose performances will be compared and discussed in Sections 3.2, 4. The first pipeline is based on the extraction of a collection of standard signal processing features commonly described in the literature. This pipeline thus constitutes a robust baseline for the comparison of other methods. The second and third pipelines are based on the estimation of covariance matrices and Riemannian geometry, after the application or not of a spatial filtering technique. This approach already demonstrated strong performances in the classification of EEG signals and is now introduced in the context of EMG classification. The fourth pipeline is based on deep learning, specifically convolutional neural networks, which have already demonstrated state-of-the-art performances in a wide range of domains. Those four pipelines are illustrated in Figure 5.

#### 2.4.1 Pipeline based on standard signal processing features

As extensively documented in the existing literature (Nazmi et al., 2016; Spiewak et al., 2018; Nguyen et al., 2021), the state-of-the-art in classifying various types of gestures using surface EMG signals has traditionally relied on features extracted through standard signal processing techniques. In this classification pipeline, we first compute the mean absolute value, root mean square, maximum absolute amplitude, waveform length, zero-crossings, slope sign changes, and maximum fractal length, as these features were some of the most reported time domain features in the literature (Oskoei and Hu, 2008; Tkach et al., 2010; Ahsan et al., 2011; Phinyomark et al., 2012; Balbinot and Favieiro, 2013; Daud et al., 2013; Al-Angari et al., 2016). Additionally, we compute the Kurtosis (Nazarpour et al., 2013) as a robust measure of signal non-Gaussianity, the Hurst exponent (Marri and Swaminathan, 2015) as a measure of chaos, or unpredictability, in the EMG signal, and Sample Entropy (Zhang and Zhou, 2012; Gao et al., 2015) as a measure of the complexity of a physiological time series (Richman and Moorman, 2000).

While it is worth noting that some of these features do capture redundant or correlated physiological characteristics, as analyzed in Phinyomark et al. (2012), we did not perform further features selection algorithm like Minimum Redundancy Maximum Relevance (MRMR) (Peng et al., 2005) or dimensionality reduction techniques like Principal Components Analysis (PCA) to decorrelate the s.

With *N* the number of time samples in the signal, *i* ∈ {1, ..., *N*} the index of a time sample, *x* ∈ ℝ^1 × *N*^ the EMG signal from one electrode, *x*_*i*_ ∈ ℝ a time sample, let $\bar{x}=\frac{1}{N}\overset{N}{\underset{i=1}{\sum }}x_{i}$ denote the mean value of *x*, $x_{\text{std}}=\sqrt{\frac{1}{N}\overset{N}{\underset{i=1}{\sum }}{\left(x_{i}-\bar{x}\right)}^{2}}$ denote the standard deviation of *x*, and sgn denote the sign function defined in Equation (1):


(1) $$\text{sgn}:\mathbb{R}\to [0,1]:\text{sgn}(x_{i}):=\{\begin{array}{ll} -1 & \text{if }x_{i}<\text{0}\\ 0 & \text{if }x_{i}\text{ = 0}\\ 1 & \text{if }x_{i}>\text{0} \end{array}$$

##### 2.4.1.1 Implementation of the pipeline

First, the features illustrated in Table 2 were computed on each electrode of the EMG signal from gesture *x* ∈ ℝ^1 × *N*^ and aggregated in a one-dimensional feature vector Ω. With *y* ∈ [0, 1] the gesture type of *x*, the final classifier of this pipeline is a logistic regression (LR) estimating the probability that a feature vector Ω must be labeled as 1 (ℙ[*y* = 1|Ω]) by the function defined in Equation (2):


(2) $$\begin{array}{l} \Lambda :\Omega \mapsto \Lambda \left(\Omega \right)=\sigma \left(\omega^{0}+\omega^{⊤}\Omega \right), \end{array}$$

for a linear classifier, where $\sigma \left(x\right)=\frac{\exp x}{1+\exp x}$ is the logistic function.

**Table 2:** Time domain features.

Mean absolute value	MAV $=\frac{1}{N}\overset{N}{\underset{1}{\sum }}|x_{i}|$
Root mean square	RMS $=\sqrt{\frac{1}{N}\overset{N}{\underset{1}{\sum }}x_{i}^{2}}$
Maximum absolute amplitude	MAA = max{|*x*_1_|, ..., |*x*_*N*_|}
Waveform length	WL $=\overset{N}{\underset{i=2}{\sum }}|x_{i}-x_{i-1}|$
Zero-crossings	ZC $=\overset{N}{\underset{i=2}{\sum }}\text{sgn}\left(-x_{i-1}x_{i}\right)$
Slope sign changes	SSC $=\overset{N}{\underset{i=3}{\sum }}\text{sgn}\left(-\left(x_{i}-x_{i-1}\right)\left(x_{i-1}-x_{i-2}\right)\right)$
Wilson amplitude	WA $=\overset{N}{\underset{i=2}{\sum }}\text{sgn}\left(|x_{i}-x_{i-1}|-x_{\text{std}}\right)$
Maximum fractal length	MFL $=\log\left(\sqrt{\overset{N}{\underset{i=2}{\sum }}{\left(x_{i}-x_{i-1}\right)}^{2}}\right)$
Kurtosis	KRT $=\frac{1}{N}\overset{N}{\underset{i=1}{\sum }}\frac{{\left(x_{i}-\bar{x}\right)}^{4}}{x_{\text{std}}^{4}}$
Hurst exponent	HUR Estimated by rescaled range procedure
	following (Alvarez-Ramirez et al., 2008)
Sample entropy	SEN Computed following (Gao et al., 2015) with parameters:
	*m* = 2, sample length = 3 and tolerance = *x*_std_/10

Preliminary experiments showed that the result of the logistic regression model was not statistically different than other classification models such as SVM, random forest, and multi-layer perceptrons. Thus, Logistic regression was chosen as the final classifier for both the time domain features and Riemannian geometry pipelines. Also, this choice was supported by the results of previous studies (Hand, 2006; Thomas et al., 2023) which showed that linear estimators can often exhibit unexpectedly strong performance when applied to real-world data.

The parameters estimated during the training of the classification pipelines are (i) the time domain features, (ii) ω^0^, ω respectively the intercept and the normal vector of the classification boundary hyperplane.

The features were computed using the Numpy (Harris et al., 2020) and SciPy (Virtanen et al., 2020) Python libraries. The multi-class logistic regression trained to classify the gesture type used the one-vs-rest training scheme with *L*^2^ penalty and *liblinear* solver, as implemented in Pedregosa et al. (2011).

#### 2.4.2 Pipelines based on covariance matrices and Riemannian geometry

In recent years, classifiers based on covariance matrices and Riemannian geometry have garnered increasing interest (Lotte et al., 2018), most notably for their first-class performance in international Brain-Computer Interface (BCI) competitions (Congedo et al., 2017). Prior studies have already provided evidence of the effectiveness of Riemannian geometry-based classification pipelines in discriminating neural signals based on covariance matrices in motor imagery (Guan et al., 2019; Majidov and Whangbo, 2019), EEG respiratory states (Navarro-Sune et al., 2017), visual evoked potential (Simar et al., 2022), and mental states (Simar et al., 2020) discrimination. While Riemannian geometry-based classification pipelines have primarily been developed and applied to brain-derived signals, such as EEG (Wu et al., 2017) and MEG (Ye et al., 2020), recent work has also used similar pipelines to classify motor control difficulty based on EMG signals (Manjunatha et al., 2020, 2022). In this work, we further demonstrate their effectiveness at discriminating EMG signals in the context of hand gesture recognition.

##### 2.4.2.1 XDAWN spatial filtering

The xDAWN algorithm, initially designed by Rivet et al. (2009), estimates spatial filters optimized in a supervised manner to enhance the signal-to-signal-plus-noise ratio (SSNR) of EEG signals related to evoked brain potentials. The xDAWN algorithm (i) first averages the EMG signals over trials per condition, then (ii) computes the noise and covariance matrices of the resulting average signals, and finally (iii) solves a generalized eigenvalue problem to obtain spatial filters that maximize the SSNR. Given that evoked brain potentials and gesture muscle potentials both exhibit spatially localized, time-locked, variations in signal amplitudes, we hypothesize that the xDAWN algorithm will demonstrate comparable effectiveness when applied to EMG signals by improving the SNR of the original EMG signal.

More formally, with *N* ∈ ℕ the total number of trials, *E* ∈ ℕ the number of electrodes, *K* ∈ ℕ the total number of gestures to estimate, *k* ∈ {1, ..., *K*} the type of gesture, *T* the number of time samples in a gesture epoch, let *P*^(*k*)^ ∈ ℝ^*E*×*T*^ denote the prototyped response, i.e. the mean EMG signal computed from all epochs of gesture type *k*, and λ ∈ ℝ^*E*×*NT*^ be the matrix obtained by concatenating all the epochs of EMG signals from the entire set of gestures. Each spatial filter is optimized to enhance the SSNR of its corresponding gesture type *k* and represented as a vector *w* ∈ ℝ^*E*×1^ defined by Equation (3):


(3) $$\begin{array}{l} w^{{*}^{\left(k\right)}}={\text{argmax}}_{w}\frac{w^{T}P^{\left(k\right)}P^{{\left(k\right)}^{T}}w}{w^{T}\lambda \lambda^{T}w} \end{array}$$

With *F* ∈ ℕ the parameterizable number of xDAWN spatial filters, let *W*^(*k*)^ denote the *F* selected spatial filters for gesture type *k*, and *W* =[*W*^(1)^, ..., *W*^(*k*)^] ∈ ℝ^*E*×*KF*^ the aggregation of those spatial filters. Let $X_{i}\in {\mathbb{R}}^{E\times T}$ a gesture epoch of index *i*, the spatially filtered signal of *X*_*i*_ is defined by $Z_{i}\in {\mathbb{R}}^{KF\times T}$ as in Equation (4):


(4) $$\begin{array}{l} Z_{i}=W^{T}X_{i} \end{array}$$

We define a new matrix ${\tilde{Z}}_{i}\in {\mathbb{R}}^{2KF\times T}$ by concatenating (i) the filtered averaged trials *P*^(*k*)^ for all gesture types *k* with (ii) the spatially filtered EMG signal *Z*_*i*_ as in Equation (5):


(5) $$\begin{array}{l} {\tilde{Z}}_{i}=\left[\begin{array}{c} W^{{\left(1\right)}^{T}P^{\left(1\right)}}\\ ...\\ W^{{\left(k\right)}^{T}P^{\left(k\right)}}\\ Z_{i} \end{array}\right] \end{array}$$

##### 2.4.2.2 Covariance matrices

In the context of EMG classification, covariance matrices capture information about how muscle signals vary together or independently. By identifying patterns and similarities in muscle activity, they provide a discriminative and compressed representation of EMG signals that have the potential to significantly enhance the performance of classification models for gesture recognition.

The set of symmetric *n* × *n* real matrices is a *n*(*n* + 1)/2-dimensional real vector space ∀*n* ∈ ℕ, and therefore has a canonical Riemannian manifold structure. Covariance matrices belong to the set of symmetric positive definite matrices which is a convex cone (Moakher, 2005; Sra and Hosseini, 2015). In such a space, the use of Euclidean distances is unsuitable due to fundamental geometric differences. The inherent curvature of Riemannian manifolds results in distances following non-linear paths, which Euclidean distances cannot accurately represent. Therefore specialized metrics or methods are needed to discriminate covariance matrices of EMG signals.

##### 2.4.2.3 Tangent space projection

In addressing this challenge, two approaches have emerged. The first involves adapting the classification models (Barachant et al., 2013; Huang and Gool, 2017), or their optimization algorithms (Kochurov et al., 2020), to accommodate the specificities of Riemannian manifolds. The second approach focuses on projecting covariance matrices from the Riemannian manifold into a specific tangent space where, given an appropriate choice of the reference point for tangent space computation, the Euclidean distance represents a good approximation of the Riemannian distance on the manifold itself (Congedo et al., 2013). The latter is particularly interesting as it enables the direct application of common classification algorithms to projected covariance matrices without significant performance loss.

Let Σ_ref_ denote the reference point on the Riemannian manifold M where the tangent plane is computed. As showed in Tuzel et al. (2008), the Fréchet mean of the set of covariance matrices is the Σ_ref_ where the projection onto the tangent space provides the better local approximation of the manifold. On this Riemannian manifold, every covariance matrices Σ ∈ M can be projected onto the tangent space computed at the reference point Σ_ref_ (Pennec et al., 2006). With *E* the number of electrodes, 1_*E*_ the matrix of ones and *I*_*E*_ the identity matrix, both of size *E*×*E*, the projection operator 𝒯_Σ_ref__ is defined by Barachant et al. (2013) as to map every matrix Σ to the vector representation of the upper triangular submatrix of $\sqrt{2}\left(1_{E}1_{E}^{T}-I_{E}\right)\Phi \left(\Sigma \right)$. With log the matrix logarithm, and log_Σ_ref__(Σ) the matrix logarithm of Σ with respect to Σ_ref_, Φ(Σ) is defined in Equation (6).


(6) $$\begin{array}{l} \Phi \left(\Sigma \right)=\log_{\Sigma_{\text{ref}}}\left(\Sigma \right)=\Sigma_{\text{ref}}^{1/2}\log\left(\Sigma_{\text{ref}}^{-1/2}\Sigma \Sigma_{\text{ref}}^{-1/2}\right)\Sigma_{\text{ref}}^{1/2} \end{array}$$

##### 2.4.2.4 Implementation of the pipelines

In this work, we evaluate the performance of two classification pipelines based on covariance matrices and Riemannian geometry.

The first pipeline called *Covariance* estimates covariance matrices $\Sigma_{i}\in {\mathbb{R}}^{E\times E}$ from $X_{i}\in {\mathbb{R}}^{E\times T}$, with *X*_*i*_ the raw EMG signal of the gesture epoch of index *i* with *E*[*X*_*i*_] = 0 as in Equation (7).


(7) $$\begin{array}{l} \Sigma_{i}=\frac{1}{N}X_{i}X_{i}^{T} \end{array}$$

The second pipeline called *xDAWN Covariance* estimates the covariance matrices $\Sigma_{i}\in {\mathbb{R}}^{2KF\times 2KF}$ from ${\tilde{Z}}_{i}\in {\mathbb{R}}^{2KF\times T}$, with *Z*_*i*_ the xDAWN-filtered EMG signal of the gesture epoch of index *i* as in Equation (8).


(8) $$\begin{array}{l} \Sigma_{i}=\frac{1}{N}{\tilde{Z}}_{i}{\tilde{Z}}_{i}^{T} \end{array}$$

Let Ω_*i*_ = 𝒯_Σ_ref__(Σ_*i*_), be the generic feature vector obtained after projecting the covariance matrix Σ_*i*_, estimated from either the raw or xDAWN-filtered EMG signal, onto the tangent space. The final classifier, common to both pipelines, is a logistic regression (LR) as defined in Equation (2).

The parameters estimated during the training of the classification pipelines are (i) the spatial filters *W* (only in the *xDAWN Covariance* pipeline), (ii) the reference point Σ_ref_ and (iii) ω^0^, ω respectively the intercept and the normal vector of the hyperplane.

The covariance matrices were computed with the well-conditioned estimator OAS (Chen et al., 2010). The estimation and application of xDAWN spatial filters, as well as the computation of Σ_ref_, and the projection operator to the tangent space 𝒯_Σ_ref__(Σ_*i*_) were implemented in the *pyRiemann* (Barachant et al., 2023) Python library. The multiclass logistic regression trained to classify the gesture type used the one-vs-rest training scheme with *L2* penalty and *liblinear* solver, as implemented in Pedregosa et al. (2011).

#### 2.4.3 Pipeline based on convolutional neural networks

##### 2.4.3.1 Network architecture

Building on the success of the perceptron (Rosenblatt, 1958) and the multilayer perceptron (Amari, 1967) (MLP) to learn complex high-dimensional patterns, new hierarchical network architectures (Fukushima, 1980) were developed inspired by previous work on the neural receptive fields of the cat visual cortex (Hubel and Wiesel, 1959). The convolutional neural network (Lecun et al., 1998) (CNN) is a biologically-inspired variant of the MLP which hierarchically extracts high-level spatial or temporal patterns using convolution operators. As of today, CNN are considered one of the state-of-the-art model for image classification and segmentation due to their first-class performance coupled with limited preprocessing requirements. These performances recently led to a growing interest from the scientific community to use CNN for the classification of physiological signals such as EEG (Dai et al., 2019) or EMG (Hioki and Kawasaki, 2009; Karnam et al., 2022).

A CNN typically consists of two main parts which play different roles in the network's architecture: the convolutional blocks and the fully-connected layers.

The convolutional blocks are responsible for feature extraction from the input data and are especially well-suited for tasks that involve grid-like data, such as images, or spatiotemporal data such as EMG. Multiple convolutional blocks are stacked to detect hierarchical features, from simple features in the early layers to more complex features in deeper layers. Each convolutional block typically comprises at least the following layers:

- A convolutional layer composed of a set of learnable *n*-dimensional kernels acting as pattern filters. Each kernel is convolved across the whole input layer to produce an activation map. Formally, in the context of a one-dimensional convolution, let *x* ∈ ℝ^*N*^ be a one-dimensional input, e.g., an EEG signal from a specific electrode, *h* ∈ ℝ^*M*^ be a one-dimensional kernel. The output of the one-dimensional convolution of *x*_*n*_ through *h*_*m*_ is given by Equation (9).
(9) $$\begin{array}{l} {\left(x*h\right)}_{n}=\overset{M-1}{\underset{m=0}{\sum }}h_{m}x_{n-m}\;\forall n=0,...,N-1 \end{array}$$
- A pooling layer which downsamples feature maps and reduces the spatial dimensions by locally applying the non-linear *max* or *mean* function.
- A regularization layer, which enhances the model's generalization and training stability by using techniques such as batch normalization or dropout. These techniques help mitigate issues like overfitting and internal covariate shift (in the case of batch normalization).

The fully-connected layers (also denoted hidden-layers) extract global patterns by combining high-level activation maps hierarchically produced by the convolutional blocks in order to make a final classification. The output of each neuron from a fully-connected layer consists of a linear combination of its inputs followed by an activation function σ. An activation function is a differentiable, non-linear function applied to the output of each neuron in order to create a non-linear decision boundary from a linear combination of inputs and weights. The use of non-linear activation functions between each hidden layer enables the separation of input vectors that are not linearly separable (Cybenko, 1989). Formally, let ℓ ∈ ℕ_>0_ denote the index of some hidden layer, $n^{\left(\ell \right)}\in {\mathbb{N}}_{>0}$ be the number of neurons in hidden layer ℓ, $z_{v}^{\left(\ell \right)}\in \mathbb{R}$ ∀*v* ∈ {1, ...*n*^(ℓ)^} be the output of the *v*^th^ neuron of layer ℓ, $w_{kv}^{\left(\ell \right)}\in \mathbb{R}$ be the weight of the edge connecting the *k*^th^ neuron of layer ℓ−1 to the *v*^th^ neuron of layer ℓ, $z_{0}^{\left(\ell \right)}\in \mathbb{R}$ be the bias of layer ℓ and σ^(ℓ)^ be the non-linear activation function of layer ℓ, the output of the *v*^th^ neuron of layer ℓ is given in Equation (10).


(10) $$\begin{array}{l} z_{v}^{\left(\ell \right)}=\sigma^{\left(\ell \right)}\left(\overset{n^{\left(\ell -1\right)}}{\underset{k=1}{\sum }}w_{kv}^{\left(\ell \right)}\;z_{k}^{\left(\ell -1\right)}+w_{0v}^{\left(\ell \right)}\;z_{0}^{\left(\ell -1\right)}\right) \end{array}$$

The final layer of the fully-connected layers is referred to as the output layer. In a classification task, the output layer typically contains one neuron per class, with the output values representing the class probabilities computed using the Softmax activation function (Bridle, 1990).

The choice of weights initialization and non-linear activation function in convolution kernels and fully-connected layers is paramount to avoid undesirable effects such as the *vanishing* or *exploding gradient* problems (Bengio et al., 1994) when training deep architectures. In this work, we used the rectified linear unit (Nair and Hinton, 2010) activation function and weights were initialized following the Glorot (Glorot and Bengio, 2010) uniform distribution. Formally, let $n^{\left(\ell \right)}\in {\mathbb{N}}_{>0}$ be the number of neurons in hidden layer ℓ, *w*^(ℓ)^ ∈ ℝ^*n*^^(ℓ−1)^×*n*^(ℓ)^ be the weights vector of layer ℓ, the initialization of *w*^(ℓ)^ following the Glorot uniform distribution is defined by Equation (11).


(11) $$\begin{array}{l} w_{ij}^{\left(\ell \right)}∽\;U\left(-\frac{\sqrt{6}}{\sqrt{n^{\left(\ell -1\right)}+n^{\left(\ell \right)}}}\;,\;\frac{\sqrt{6}}{\sqrt{n^{\left(\ell -1\right)}+n^{\left(\ell \right)}}}\right) \end{array}$$

##### 2.4.3.2 Proposed CNN architecture

After trying multiple network architectures, the following architecture achieved the best cross-validated performance both for phasic and tonic EMG:

- A linear temporal convolution with 128 filters of shape (1 × 16).
- A spatial convolution with 16 filters of shape (8 × 1) followed by a batch normalization layer, a ReLU non-linear activation function, a mean pooling layer of shape (1 × 4) and a spatial dropout layer.
- A temporal convolution with 128 filters of shape (1 × 16) followed by a batch normalization layer, a ReLU non-linear activation function, a mean pooling layer of shape (1 × 4) and a spatial dropout layer.
- Two fully-connected layers of 128 neurons each followed by a ReLU non-linear activation function.
- A fully-connected layer of four neurons (corresponding to the 4 different gestures) followed by a softmax activation function.

##### 2.4.3.3 Network training procedure

The training procedure of the CNN relies on the backpropagation algorithm (Rumelhart et al., 1986), which vanilla implementation is based on stochastic gradient descent. The backpropagation algorithm iteratively updates the set of trainable parameters of the CNN by using the chain rule to compute the partial derivatives of the loss function with respect to those parameters. With θ_*t*_ the parameters at time step *t*, α the fixed learning rate, and ∇*f*(θ_*t*_) the gradient of the loss function with respect to θ_*t*_. The updated parameters are defined by Equation (12).


(12) $$\begin{array}{l} \theta_{t+1}=\theta_{t}-\alpha \nabla f\left(\theta_{t}\right) \end{array}$$

In this work, we use the Adam optimization algorithm (Kingma and Ba, 2014) that enhances the traditional stochastic gradient descent by accelerating its convergence using adaptive learning rates for each parameter, incorporating momentum and adaptive step sizes. The starting learning used in the proposed architecture is 0.005.

### 2.5 Validation methodology

**Figure 6:**
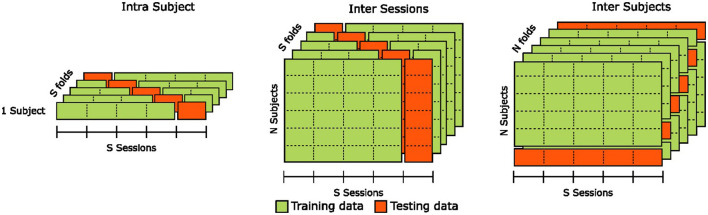
Illustration of the cross-validation methods used for intra-subjects, inter-sessions, and inter-subjects analyses of classification results.

To validate the classification results, we evaluate the generalization capabilities of the estimation pipelines in three distinct configurations illustrated in Figure 6: the intra-subjects, inter-sessions, and inter-subjects.

In the intra-subjects configuration, we assess the estimator's robustness by training it on 80% of the training set and evaluating it on the remaining 20%, representing a “holdout" portion of the data that the model has never seen. To prevent potential bias due to specificities in portions of the training set or uneven class representation in the validation set, we use a stratified five-fold cross-validation and report the mean accuracy across all folds. We repeat this procedure for each participant, and the final estimator performance is computed as the mean accuracy across all individual cross-validated estimators. The intra-subjects configuration provides insights into an estimator's capacity to generalize to new, unseen data from the same participant. However, it does not provide any information on the estimator's ability to generalize to data from different participants.

In the inter-sessions configuration, we assess the estimator's robustness by training it on four out of the five sessions of all participants and evaluating it on the “holdout” sessions that the model has never seen. To prevent bias due to potential sessions' specificities, we use a Leave-One-Group-Out cross-validation, with each “group” corresponding to a session. The final estimator performance is computed as the mean accuracy across all five cross-validated estimators. The inter-sessions configuration provides insights into an estimator's capacity to generalize to unseen data from new sessions of the same participants. However, similarly to the intra-subjects configuration, it does not provide any information on the estimator's ability to generalize to data from new unseen participants.

In the inter-subjects configuration, we assess the estimator's robustness by training it using data from all but one participants and evaluating it on the “holdout" participant that the model has never seen. To prevent bias due to potential participants' specificities, we use a Leave-One-Subject-Out cross-validation. The final estimator performance is computed as the mean accuracy across all *n* cross-validated estimators, with *n* being the number of participants. Contrary to intra-subjects and inter-sessions configurations, the inter-subjects configuration provides insights into an estimator's capacity to generalize to unseen data from new participants.

### 2.6 Physiological decomposition of the EMG signal

As previously introduced, the EMG signal can be categorized as phasic (during the production of movement) or tonic, during steady postures. Defining the precise boundary between these categories can be challenging. In this study, we decided to distinguish between tonic and phasic activity by choosing a specific time period after a careful visual inspection of the EMG signals. Another interesting perspective is to consider EMG signals as a combination of two commands: the “move command” that controls the movement of the limb to the target posture and influences primarily phasic activity, and the “hold command” that maintains the limb at the target posture and influences tonic activity. The raw EMG signal represents the sum of both commands.

How to decompose the signal into “move” and “hold” commands is not completely understood yet. Albert et al. (2020) have hypothesized that, for hand gestures, the instantaneous amplitude of the “hold” command of a specific posture is defined by the integral of the instantaneous amplitude of the “move” command that led to this posture. The instantaneous amplitude of the EMG signal (also referred to as the “envelope” of the signal) represents the strength of the muscle activation at any point in time, regardless of the phase of the signal.

We compute the envelope of the EMG signal by initially performing full-wave rectification on the analytic signal derived from the application of the Hilbert transform (Boashash, 1992; Myers et al., 2003). Formally, for a given signal *x*(*t*), we have its instantaneous amplitude $a\left(t\right)=x^{2}\left(t\right)+x_{h}^{2}\left(t\right)$, where *x*_*h*_(*t*), the Hilbert transform of *x*(*t*), is computed using the formula in Equation (13).


(13) $$\begin{array}{l} x_{h}\left(t\right)=p.v.\int_{-\infty }^{+\infty }\frac{x\left(t-\tau \right)}{\pi \tau }d\tau \end{array}$$

where *p*.*v*. denotes the Cauchy principal value of the integral (Boashash, 1992). Finally, we apply low-pass filtering with a 20 Hz cutoff frequency to smooth the resulting envelope.

Let us note *H*_*T*_, the instantaneous amplitude of the hold command at time *T*, and *M*_*T*_ the instantaneous amplitude of the move command at time *T*. The mathematical integration of physiological signals described by Albert et al. (2020) is as follows in Equation (14).


(14) $$\begin{array}{l} H_{T}=\int_{0}^{T}M_{t}dt \end{array}$$

In the envelope of a discrete recorded EMG signal *S*, the value at each time step *T* is equal to the sum of the two commands: *S*_*T*_ = *M*_*T*_+*H*_*T*_. To estimate the decomposition of the signal's envelope into *M* and *H*, we use the iteration defined in Equation (15).


(15) $$\{\begin{array}{l} M_{T}=S_{T}-H_{T-1}\\ H_{T}=H_{T-1}+\frac{M_{T}}{f} \end{array}$$

In these formulas, *f* is the sampling frequency. Initially, *H*_0_ and *M*_0_ are set to 0 and *T* = 0 should correspond to the beginning of the move command. After decomposing the signal, we obtain two separate envelopes, that can be used as input of a machine learning model, similarly to the raw signal.

We note that the mathematical integration method is not the only hypothesis for decomposing the EMG signal into its fundamental components. For example, Flanders and Soechting (1990) described a more complex analysis based on principal components scaled down with movement time.

## 3 Results

### 3.1 EMG visualization during guided gestures

**Figure 7:**
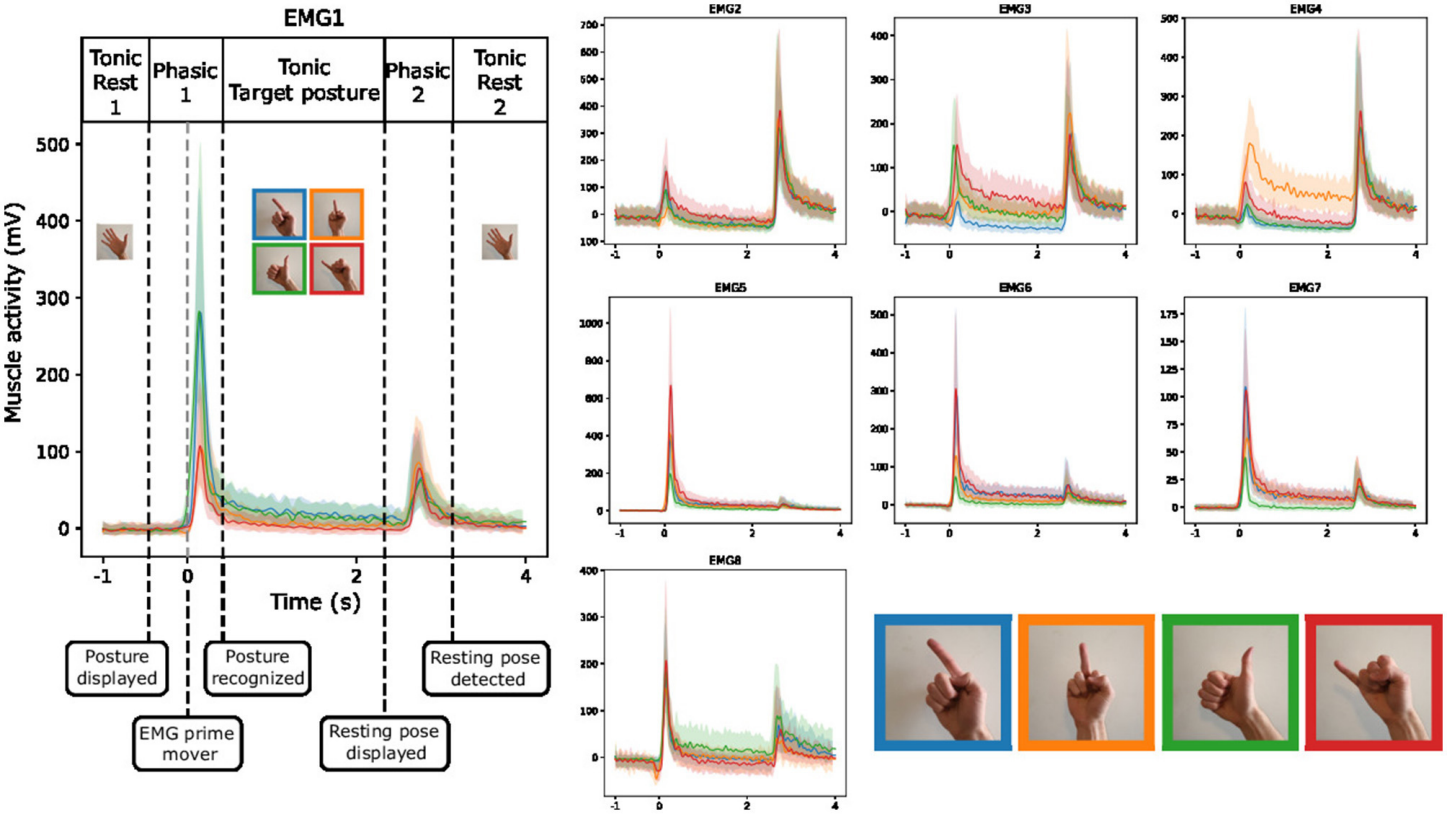
Envelope (median and quartiles) of the EMG signal during the realization of predefined gestures by one subject (right hand of the subject 4). The signals are aligned at the prime mover event.

We aim to gain a comprehensive understanding of the EMG behavior during guided gestures. To achieve this, we present an analysis of the median and quartile values of the envelope of the EMG signal (as presented in Section 2.6) during the execution of the four distinct gestures. We also remove the baseline activity associated with the resting pose, thereby allowing us to highlight the dynamic aspects of muscle activity during the guided gestures. As shown in Figure 7, the gestures involve three phases. First, when the subject transitions from the resting pose to the target pose, we observe a short and strong increase in muscle activity. After that, a residual activity is observed during the hold of the posture, indicating that the muscles are still more engaged than during the resting posture. Finally, a second peak of activity appears when going back to the resting pose. The plots of different EMG channels in Figure 7 indicate a clear difference between the roles of each muscle. Those more active during the first phase might correspond to flexor muscles, while those more active during the last phase should be extensor muscles.

Figure 7 suggests that discriminating between the recorded gestures executed by a single participant's hand should be a manageable task, given the distinctive EMG patterns exhibited by the different gestures. Especially, when looking at the EMG3 channel, we observe strong differences between gestures during both the first gesture phasic and the tonic phase of the target posture.

**Figure 8:**
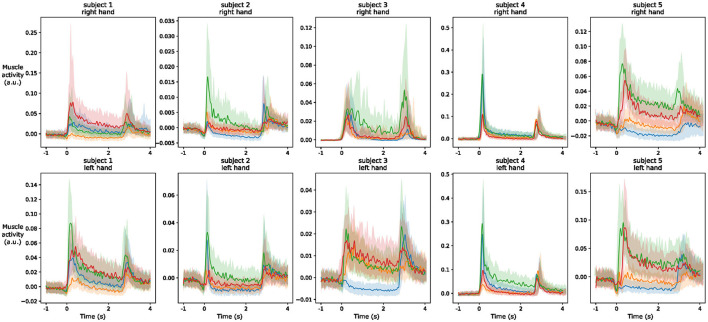
Envelope (median and quartiles) of the EMG signal of electrode 1 during the realization of predefined gestures by different subjects. The signals are aligned at the prime mover event and normalized using division by the maximum value of the signal during maximum voluntary contraction of the muscles. The colors of the four gestures are similar to those in Figure 7.

To better understand inter-subjects variability, we use a similar figure that highlights the behavior of the EMG1 channel on the two hands of different participants. We observe from Figure 8 that the overall amplitude of the signal changes significantly across the subjects even with normalization applied from MVC. Moreover, regardless of the signal amplitude, the shape of the signal during the different gestures is also subject-dependent, and often even changes between the left and right hand of the same person. These differences help to understand the lower efficiency of inter-subject estimation models that are commonly obtained in the literature.

### 3.2 Classification of guided gestures

**Figure 9:**
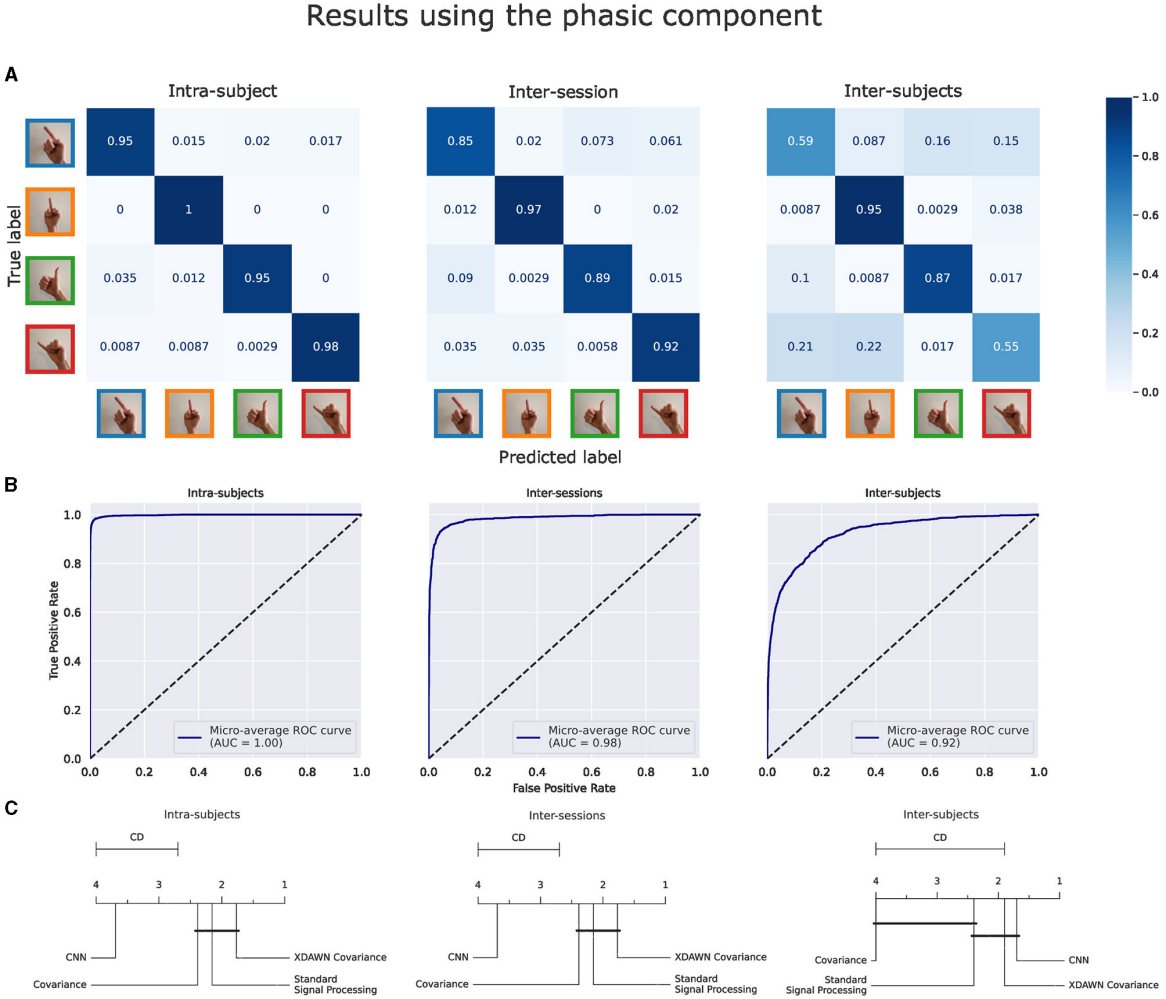
Results of the voting ensemble of the four classification pipelines using the phasic component of the EMG signals in intra-subject, inter-sessions, and inter-subjects configurations. **(A)** Confusion matrices. **(B)** ROC curves. **(C)** Average rank of the corresponding classifiers (the lower, the better). Classifiers that are not significantly different are connected by a black line [at *p = 0.05* found by a Nemenyi test (Demšar, 2006)]. The critical distance (CD) indicates when classifiers are considered statistically different.

**Figure 10:**
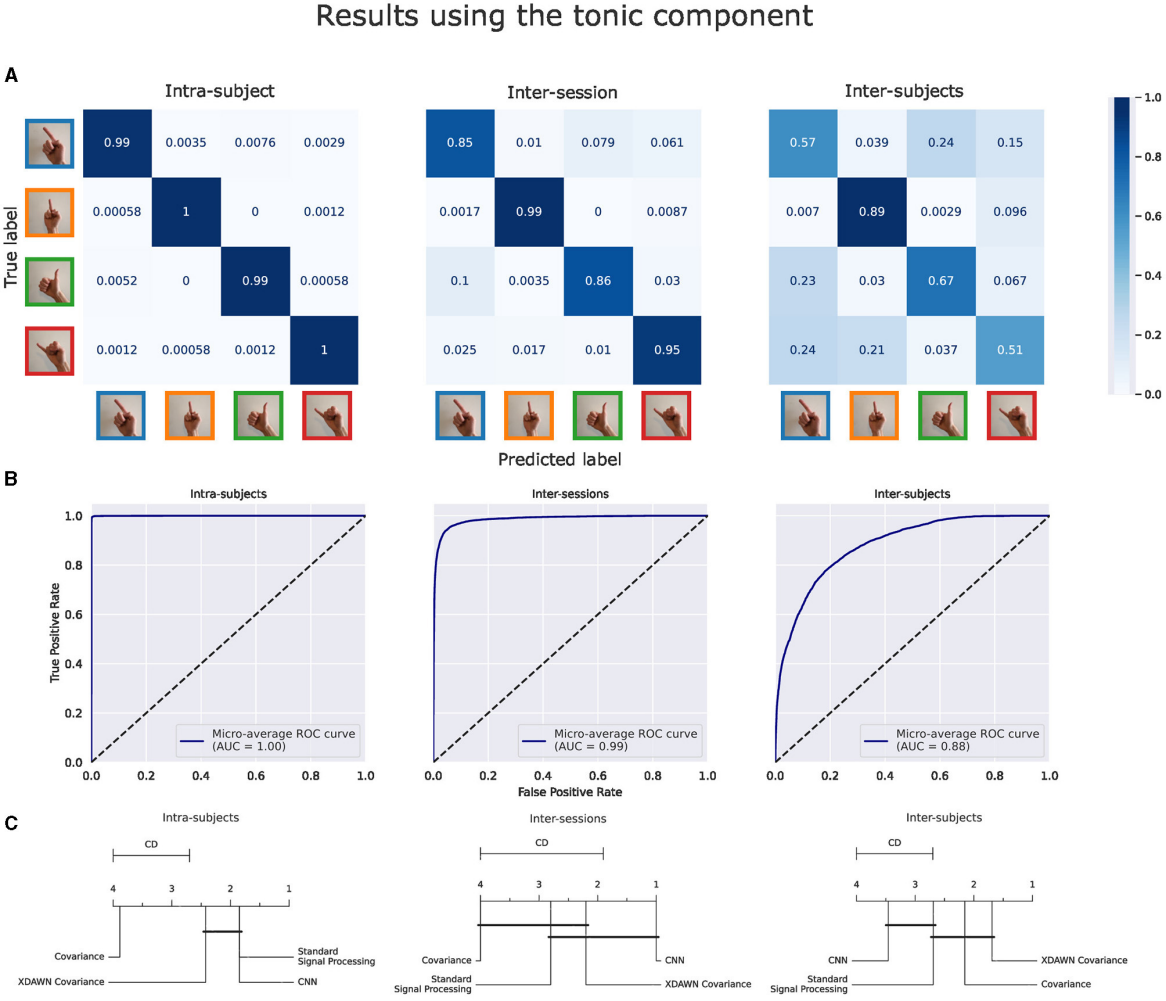
Results of the voting ensemble of the four classification pipelines using the tonic component of the EMG signals in intra-subject, inter-sessions, and inter-subjects configurations. **(**A) Confusion matrices. **(B)** ROC curves. **(C)** Average rank of the corresponding classifiers (the lower, the better). Classifiers that are not significantly different are connected by a black line [at *p = 0.05* found by a Nemenyi test (Demšar, 2006)]. The critical distance (CD) indicates when classifiers are considered statistically different.

**Figure 11:**
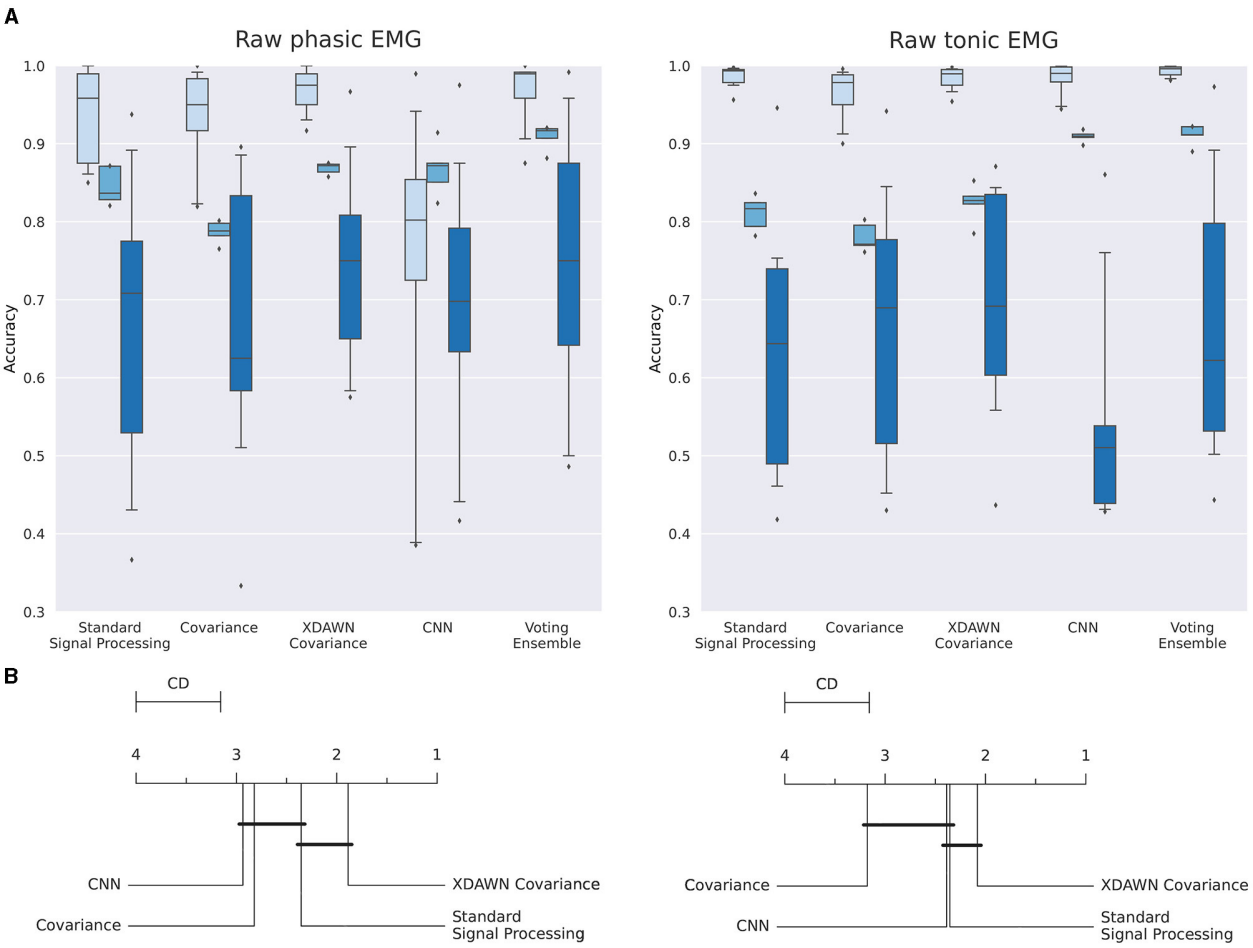
**(A)** Boxplots illustrating the multiclass classification accuracy of the four classification pipelines and the Voting ensemble using the phasic or tonic components of the EMG signals in intra-subject (light blue), inter-sessions (medium blue), and inter-subjects (dark blue) configurations. **(B)** Average rank of the corresponding classifiers for the three configurations (the lower, the better). Classifiers that are not significantly different are connected by a black line [at *p = 0.05* found by a Nemenyi test (Demšar, 2006)]. The critical distance (CD) indicates when classifiers are considered statistically different.

The main results of the classifications pipelines using both the phasic and tonic components of EMG signals in intra-subject, inter-sessions, and inter-subjects configurations were illustrated by confusion matrices, receiver operating characteristic (ROC) curves (with the corresponding AUC values), and average ranks of the classification pipelines. For the clarity of illustration, we aggregated the results from the four classification pipelines by computing the average of their predictions and denoted this new model *Voting ensemble*. The aggregated performances of the four classification pipelines using the phasic and tonic components are illustrated in Figures 9, 10 respectively. The individual performances of each classification pipeline are illustrated in Figure 11. Statistical differences between classification pipeline performances were performed with a *post-hoc* Nemenyi test (Demšar, 2006) using the Autorank Python library (Herbold, 2020).

Additionally, to assess the performances of the four classification pipelines using high-quality EMG signals, we excluded the sessions with low-quality EMG (as explained in Section 2.3) from the dataset used to generate the results of the present section.

#### 3.2.1 Classification results using the phasic component of the EMG signals

Figure 9 illustrates these performance metrics computed on the Voting ensemble in intra-subject, inter-sessions, and inter-subjects configurations using only the phasic component of the EMG signals. The Voting ensemble model achieved an accuracy of 96.7%, 90.9%, and 73.9%, along with AUC values of 1.0, 0.98, and 0.92, in the intra-subjects, inter-sessions, and inter-subjects configurations, respectively. The confusion matrices in Figure 9A show a balanced recognition accuracy in the intra-subjects configuration but not in the inter-subjects configuration, where gestures involving the index and pinky fingers were notably less well classified than those involving the thumb and middle fingers. The average ranks illustrated in Figure 9C show that the xDAWN Covariance pipeline achieved the highest rank among the four classification pipelines in the intra-subjects and inter-sessions configurations. However, Figure 9C did not show a significant statistical difference between the performances of xDAWN Covariance and the other best-performing classification pipelines. Interestingly, while the CNN pipeline exhibited significantly lower performance than the other classification pipelines in intra-subjects (likely due to the limited amount of data from the small number of gesture repetitions per subject) and inter-sessions configurations, it ranked highest in the inter-subjects configuration, slightly ahead of the xDAWN Covariance pipeline.

#### 3.2.2 Classification results using the tonic component of the EMG signals

Figure 10 illustrates the same performance metrics as Figure 9 computed on the Voting ensemble in intra-subject, inter-sessions, and inter-subjects configurations but using only the tonic component of the EMG signals. The Voting ensemble model achieved an accuracy of 99.3%, 91.1%, and 66.7%, along with AUC values of 1.0, 0.99, and 0.88, in the intra-subjects, inter-sessions, and inter-subjects configurations, respectively. Similarly to the confusion matrices from the phasic component, the confusion matrices from the tonic components in Figure 9A show a balanced recognition accuracy in the intra-subjects configuration but not in the inter-subjects configuration, where, gestures involving the index and pinky fingers were notably less well classified than those involving the thumb and middle fingers. The average ranks illustrated in Figure 9C did not highlight a particular significant statistical difference between the performances of the four classification pipelines using the tonic component of the EMG signals. Here, the CNN pipeline in the intra-subjects configuration exhibits significantly better performance using the tonic component.

#### 3.2.3 Summary of the classification results

The individual performances of each classification pipeline using both the phasic and tonic components of EMG signals in intra-subject, inter-sessions, and inter-subjects configurations are summarized and illustrated in Figure 11.

Figure 11A (left, right) illustrate the individual performances using respectively the phasic and the tonic components of the EMG signals in intra-subject, inter-sessions, and inter-subjects configurations.

In Figure 11A (left), in the intra-subjects, inter-sessions, and inter-subjects configurations, respectively, the classification pipeline based on standard signal processing features achieved an accuracy of 94.1%, 84.6%, and 67.7%; the Covariance pipeline achieved an accuracy of 93.5%, 78.7%, and 66.5%; the xDAWN Covariance pipeline achieved an accuracy of 96.9%, 86.8%, and 73.9%; the CNN pipeline achieved an accuracy of 75.3%, 86.7%, and 69.2%; and the Voting ensemble achieved an accuracy of 96.7%, 90.9%, and 73.9%.

In Figure 11A (right), in the intra-subjects, inter-sessions, and inter-subjects configurations, respectively, the classification pipeline based on standard signal processing features achieved an accuracy of 98.7%, 81.1%, and 62.8%; the Covariance pipeline achieved an accuracy of 96.6%, 78.0%, and 65.8%; the xDAWN Covariance pipeline achieved an accuracy of 98.3%, 82.3%, and 68.9%; the CNN pipeline achieved an accuracy of 98.4%, 90.9% and 54.2%; and the Voting ensemble achieved an accuracy of 99.3%, 91.1%, and 66.7%.

With the average ranks computed for each pipeline based on the aggregation of their respective predictions from the intra-subjects and inter-sessions configurations, Figure 11B shows that the xDAWN Covariance pipeline achieved the highest rank among the four classification pipelines using either the phasic or the tonic component. The xDAWN Covariance pipeline achieved significantly higher performances than the CNN and the Covariance pipeline using the phasic component, as well as the Covariance pipeline using the tonic component. However, Figure 11B did not show a significant statistical difference between the performances of the xDAWN Covariance pipeline and the pipeline based on standard signal processing.

### 3.3 Physiological decomposition of EMG for classification

**Figure 12:**
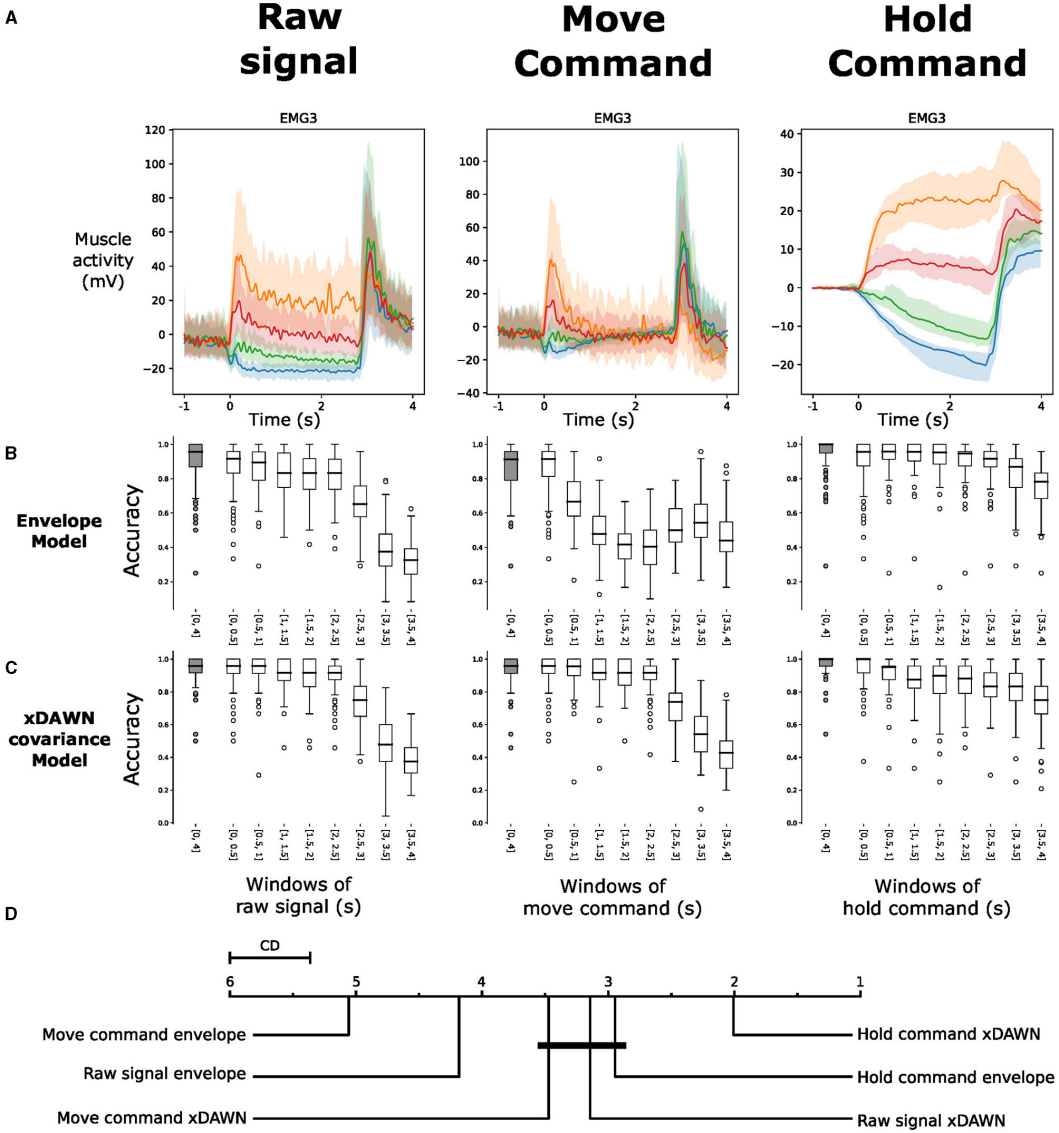
**(A)** Visualization of the EMG commands during four hand gestures on different muscles from one subject with a decomposition of the recorded signal into move and hold commands. The colors of the four gestures are similar to those in Figure 7. **(B)** Box plots of the intra-subject accuracy obtained with a logistic regression model directly on the signal envelope in the different components. **(C)** Box plots of the intra-subject accuracy obtained with a logistic regression model on the xDAWN covariances matrices from the different components. **(D)** Average rank of the corresponding classifiers (the lower, the better). Classifiers that are not significantly different are connected by a black line [at *p = 0.05* found by a Nemenyi test (Demšar, 2006)]. The critical distance (CD) indicates when classifiers are considered statistically different.

Decomposing the signal into its fundamental components could potentially help to recognize the gesture by incorporating physiological knowledge into the machine learning model. Figure 12 illustrates the physiological decomposition of the EMG signal into the “move" and “hold" commands, as described by Albert et al. (2020). The signals obtained from the mathematical integration have a physiologically plausible shape. The move command contains short bursts of activity during the gestures and is close to zero during the posture hold. The hold command quickly increases during the gesture, then stays mostly flat during the hold. As we continued to integrate the signal during the second gesture, leading back to the resting pose, a second increase of the signal was found in the extracted hold command. This part of the integrated signal has no physiological interpretation from Albert et al. (2020). However, we can see a higher difference between the classes in the integrated signal than in the raw signal even after the second gesture. Hence, we kept this part of the integrated signal in the extracted hold command to assess the performance of classification models.

We compare classification models on different parts of the three signals. For each window of 0.5 s between *T* = 0*s* and *T* = 4*s*, we test a classification model to see the importance of each part of the different components of the signal. We also assess the performance of a model that takes the complete gesture (from *T* = 0*s* to *T* = 4*s*) as input. We use two classification models. The first is a logistic regression classifier trained using the signal envelope. The second is an xDAWN Covariance classification pipeline, as it achieved the best classification results in Section 3.2.

We expect that the first model is more sensitive to the differences in the amplitude of the signal, whereas the second model might be sensitive to both the signal's amplitude, variance and covariance. As the results in Section 3.2 showed that intra-subject classification is already near-perfect when sessions with low signal quality are removed, we performed this classification using all sessions, regardless of signal quality, to highlight the value of the present method. The results of those two models are shown in Figures 12B, C.

Figure 12 shows that both the move and the hold commands contain relevant information during the whole gesture for the classification. With the xDAWN Covariance pipeline, it is even possible to classify the held posture during the holding phase using only the move command. The accuracy only drops when the subject stops holding the specific posture and goes back to the resting pose. Also, we see that with both kinds of features, extracting the hold command enabled us to increase the best classification result compared to the raw signal. In particular, when using the xDAWN Covariance pipeline on the whole time range (from 0 to 4 s), the accuracy with the raw signal is 94.7 ± 0.08% whereas, the accuracy with the hold command is 97.7 ± 0.06%. As shown by the statistical analysis in Figure 12D, this result is significantly better than the results obtained by all the others models. Interestingly, when using 0.5 s windows of signal, the model with the best results uses the xDAWN Covariance pipeline trained with the hold command during the gestures (between 0 and 0.5 s). This window corresponds to the rapid change in the amplitude of the signal at the beginning of the hold command, where the variance of the hold command is the highest.

**Figure 13:**
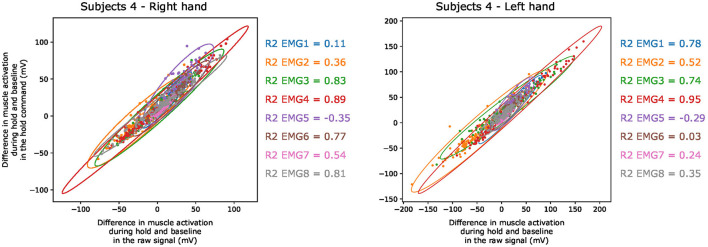
Changes in muscle activation between the hold periods of the resting pose and the target posture in the raw signal Vs in the hold command extracted using the mathematical integration method. The two hands of one subject are reported as examples, and different colors are used to represent the EMG channels. The R2 scores are reported for each channel.

To validate the mathematical integration hypothesis for extracting move and hold commands, we compute the person correlation coefficients and R2 scores between the amplitude of the extracted hold command and the raw signal during the posture hold. We computed these scores separately for each EMG channel of each participant and obtained and obtained an average R2 of 0.697 ± 0.348 and an average correlation coefficient of 0.937 ± 0.066. In Figure 13, we show a representation of this similarity to the ones used by Albert et al. (2020) and report the R2 score for each channel of the two hands from one subject. We find that for most of the channels, there is a high similarity between the amplitude of the raw signal during the posture hold and the amplitude of the hold command at the same time. For the channels where we obtain a low or negative R2 score, we still observe a high correlation coefficient. This occurs when the values obtained in the hold command are different from those in the tonic raw signal by a constant factor.

## 4 Discussion

In recent years, there have been various initiatives to publish datasets of EMG signals and pose estimations (Atzori et al., 2014; Lobov et al., 2018; Jarque-Bou et al., 2019; Pradhan et al., 2022) aiming to provide the essential data required for improving machine learning models, myoelectric prostheses control, and, ultimately, restoring natural hand function for people with upper-limb disabilities. Typically, these datasets use either a clinical or consumer-grade EMG signals acquisition system with 8–12 EMG electrodes and a Cyberglove^4^ for hand motion capture.

In this work, we introduce a novel experimental paradigm for acquiring EMG signals and hand motion capture data during guided bimanual activities in virtual reality. This paradigm enables the automated and precise recording of multiple repetitions of various gestures using affordable and easy-to-use motion capture equipment. We believe that the automated data recording method proposed in this experimental paradigm is a critical factor for increasing movement repetitions and accelerating post-processing when compared to traditional approaches in the literature. Using virtual reality, in contrast to the Cyberglove alternative, enables a straightforward 3D visualization of motion capture data through the Unity framework (Simar, 2023).

In neuroscience, and more broadly in electrophysiology, inter-subjects variability has been a challenge for decades and remains a salient research topic (Colot et al., 2023). In this study, this challenge was highlighted by the significant variations in signal amplitude among subjects, which persisted even after MVC normalization. Moreover, the shape of the EMG signal during the four gestures also exhibits inter-subjects variability due to intrinsic variations in anatomical characteristics among subjects, possibly combined with slight shifts in electrode placement. More practically, the inter-subjects difficulty is also illustrated by a significant drop of 22.8 and 32.6% in classification accuracy between the intra-subjects and inter-subjects configurations, using the phasic and tonic components of the EMG signals, respectively. While myoelectric prostheses are expected to be tailored to each patient to account for individual anatomical characteristics, the development of new methods capable of mitigating inter-subject variability could reduce the need for frequent recalibration through numerous repetitions of specific gestures. This observation highlights the significance of developing novel techniques and models capable of generalizing to new data distributions, including those from new subjects or existing subjects with variations in electrode placement. Such capabilities are critical for myoelectric prostheses to maintain accurate functionality across various situations and environments.

Deep learning models have demonstrated their performance in various domains, including image recognition (Li, 2022), bioinformatics (Zhang et al., 2020), natural language processing (Hu, 2020), and ecology (Christin et al., 2019). Provided that such deep architectures are trained with sufficient data, they often reduce reliance on expert knowledge for data preprocessing and enhance state-of-the-art results. Here, despite recording more or a comparable number of gesture repetitions (30 repetitions of each of the four postures) than other publicly available datasets, Figure 11A shows that, in the intra-subject configuration, the proposed convolutional neural networks failed to converge for each participant, achieving only 75.3% accuracy using the 30 repetitions of phasic components. This is significantly lower than the 98.4% accuracy after training with the 150 windows of tonic components. These results further emphasize the need for an experimental paradigm which enables the recording of an increased number of movement repetitions, particularly when considering the use of deep learning architectures.

In contrast, Figure 11A shows the effectiveness of the classification pipeline based on xDAWN spatial filtering and covariance matrices with Riemannian geometry, even with a limited number of gesture repetitions. As no single classification pipeline outperformed the others consistently and significantly, we hypothesize that combining them with a meta estimator could lead to further improvements of classification performance. Interestingly, recent approaches (Chen et al., 2022) have also suggested combining the discriminative capabilities of covariance matrices with convolutional neural networks (Huang and Gool, 2017) trained with Riemannian optimizers (Kochurov et al., 2020).

Another promising yet largely unexplored path for improving performance involves integrating physiological knowledge into either signal preprocessing or machine learning models. As an example of the latter, Draye et al. (2002) demonstrated that differentiating between inertial and postural activities improved the ability of a neural network to identify mapping relationships between EMG signals and limb trajectories during complex movements.

David Robinson first introduced the idea of a neural integrator in the field of eye movement control (Robinson, 1968). He hypothesized that a group of neurons could perform mathematical integration on phasic commands to generate accurate eye positions, stabilizing retinal images during ocular movements (Chéron et al., 1986; Cannon and Robinson, 1987). Building upon the idea from Albert et al. (2020) that the neural integrator can extend to general motor control (Klier et al., 2002; Cheron et al., 2023), we found that the envelope of the rectified EMG signal during the hold command not only corresponds to the mathematical integration of the envelope during the move command but also contains significant discriminative potential.

To explore this hypothesis further, we extracted the hold command using the mathematical integration method proposed in Albert et al. (2020), closely aligned with decades of research on the neural integrator (Seung et al., 2000; Koulakov et al., 2002; Gupta and Shaikh, 2020). We obtained high correlation coefficients and R2 scores, showing the similarity in signal amplitude between the tonic activity in the raw signal and the hold command during the hand posture. Figure 12 indicates that the integral accurately extracts the hold information contained in the signal amplitude. This observation is supported by the decrease in accuracy between 0.5 and 2.5 s when using the envelope of the move command as input for the classification model (Figure 12B). Yet, during the posture hold, the xDAWN Covariance pipeline continues to achieve a high classification accuracy using the move command as input (Figure 12C). This result notably demonstrates that the information in the EMG signal related to holding a specific posture is not only encoded in the amplitude of the signal but also in its variance.

With this technique, combined with xDAWN spatial filtering, we improved the intra-subjects accuracy of the xDAWN Covariance pipeline on the complete gesture from 94.7 ± 0.08% to 97.7 ± 0.06%. In the inter-subjects configuration, the xDAWN Covariance pipeline also achieved improved classification accuracy for both the phasic and tonic components of the EMG signals. These results strongly support the hypothesis that incorporating physiological knowledge into machine learning models or signal preprocessing techniques (such as spatial filtering or physiological integration) holds the potential to significantly improve gesture recognition.

These findings could be leveraged in future research to enhance machine learning models for prosthesis control, particularly for sustained gesture recognition. To further extend the introduction of neurophysiological knowledge to machine learning models, future research might also consider the compatibility of the neural integrator with different types of feedback. Particularly in prosthesis control, we should consider the role of proprioceptive and visual feedback, which could positively contribute to the elaboration of the tonic signal (Seung et al., 2000).

### 4.1 Limitations toward myoelectric prosthesis control

While the results presented in this study demonstrate that machine learning models can almost perfectly decode simple hand gestures from EMG signals in the intra-subject configuration, caution should be exercised when translating these findings to myoelectric prosthesis control.

This experimental paradigm using virtual reality does not capture the execution of grasping tasks involving physical objects. Consequently, machine learning models may not account for the forces applied by users when manipulating physical objects. To address this limitation, we suggest replacing the VR headset with an augmented reality headset, which can simultaneously perform hand tracking while users safely interact with physical objects.

Since our dataset does not include subjects with disabilities, this study does not demonstrate the generalizability of its results to this population. Nevertheless, the techniques presented in this work can also be applied for gesture estimation in people without upper-limb disabilities, such as in sign language recognition.

In out-of-the-lab environments, EMG signals will be contaminated by non-physiological artifacts, which likely reduces the decoding performance of myoelectric prostheses. In addition, prostheses users perform unconstrained movements, without predefined triggers to determined gesture timing. Therefore, machine learning models must evolve to accurately recognize intended gestures in real time. In this context, separating EMG signals into “move" and “hold" commands can be more challenging. To pave the way to a more natural control of myoelectric prostheses, machine learning models should be able to decode full hand poses during the execution of unconstrained movements. While the novel experimental paradigm presented in this study enables the recording of the full hand poses alongside EMG signals, addressing this challenging task was beyond the scope of the study.

## 5 Conclusion

In this work, we first presented a novel experimental paradigm for the acquisition of EMG signals and hand motion capture data during guided bimanual activities in virtual reality. Using the data recorded from 14 healthy participants, we compared the performance of multiple state-of-the-art classification pipelines for gesture recognition based on both the phasic and tonic components of the EMG signals. We introduced and demonstrated the performance of classification pipelines based on covariance matrices with Riemannian geometry for EMG classification, achieving 96.9%, 86.8%, and 73.9% accuracy using the phasic component, and 98.3%, 82.3%, and 68.9% accuracy using the tonic component in intra-subjects, inter-sessions, and inter-subjects configurations, respectively. Using state-of-the-art classification pipelines, we also showed that the tonic component contains comparable discriminative power to the phasic component for gesture recognition.

We showed that introducing physiologically informed feature extraction to classification pipelines can further improve the efficiency of hand gesture recognition. Specifically, the introduction of the neurophysiological integration of the “move command” improved the performances of the state-of-the-art classification algorithm by 3% when predicting the “hold command.”

In the context of human assist devices, the various contributions of this study notably imply that robust prosthesis control should rely on both phasic and tonic components for the continuous recognition of a desired posture and that their decoding performance may be further enhanced by the integration of fundamental neurophysiological methods.

## Data availability statement

The raw data supporting the conclusions of this article will be made available by the authors, without undue reservation.

## Ethics statement

The studies involving humans were approved by Ethics Committee of Université Libre de Bruxelles, CHU Brugmann. The studies were conducted in accordance with the local legislation and institutional requirements. The participants provided their written informed consent to participate in this study.

## Author contributions

CS: Conceptualization, Data curation, Formal analysis, Investigation, Methodology, Software, Supervision, Validation, Visualization, Writing – original draft, Writing – review & editing. MC: Data curation, Formal analysis, Methodology, Software, Validation, Visualization, Writing – original draft, Writing – review & editing. A-MC: Methodology, Supervision, Writing – review & editing. MP: Data curation, Project administration, Resources, Software, Writing – review & editing. GC: Conceptualization, Formal analysis, Funding acquisition, Methodology, Supervision, Writing – review & editing. GB: Conceptualization, Funding acquisition, Supervision, Validation, Writing – review & editing.

## Footnotes

^1^ https://www.meta.com/quest/

^2^ https://unity.com/

^3^ https://docs.unity3d.com/Manual/VROverview.html

^4^ http://www.cyberglovesystems.com
